# Meta-analysis of incidence of early lung toxicity in 3-dimensional conformal irradiation of breast carcinomas

**DOI:** 10.1186/1748-717X-8-268

**Published:** 2013-11-14

**Authors:** Gokula, Arul Earnest, Lea Choung Wong

**Affiliations:** 1 Advanced Medical and Dental Institute, Universiti Sains Malaysia, Penang, Malaysia; 2 Department of Radiation Oncology, National University Cancer Institute, Singapore, Singapore; 3 William Buckland Radiotherapy Centre, Alfred Health, Melbourne, Australia; 4 Centre for Quantitative Medicine, Office of Clinical Sciences, Duke-NUS Graduate Medical School, Singapore, Singapore

**Keywords:** Breast radiotherapy, Radiation pneumonitis, Lung toxicity, Meta-analysis

## Abstract

**Background:**

This meta-analysis aims to ascertain the significance of early lung toxicity with 3-Dimensional (3D) conformal irradiation for breast carcinomas and identify the sub-groups of patients with increased risk.

**Methods:**

Electronic databases, reference sections of major oncological textbooks and identified studies were searched for synonyms of breast radiotherapy and radiation pneumonitis (RP). Major studies in thoracic irradiation were reviewed to identify factors frequently associated with RP. Meta-analysis for RP incidence estimation and odds ratio calculation were carried out.

**Results:**

The overall incidence of Clinical and Radiological RP is 14% and 42% respectively. Ten studies were identified. Dose-volume Histogram (DVH) related dosimetric factors (Volume of lung receiving certain dose, V_dose_ and Mean lung Dose, MLD), supraclavicular fossa (SCF) irradiation and age are significantly associated with RP, but not sequential chemotherapy and concomitant use of Tamoxifen. A poorly powered study in IMN group contributed to the negative finding. Smoking has a trend towards protective effect against RP.

**Conclusion:**

Use of other modalities may be considered when Ipsilateral lung V_20Gy_ > 30% or MLD > 15 Gy. Extra caution is needed in SCF and IMN irradiation as they are likely to influence these dosimetric parameters.

## Introduction

Postoperative radiotherapy (RT) after the breast conservative surgery (BCT) or mastectomy, have been shown to reduce the rates of local recurrence and death in breast carcinomas (BC) [1-5]. It is the standard practice to offer patients adjuvant RT to whole breast or chest wall, with or without loco-regional RT (LRRT), depending on the stage of disease. The regional fields include the ipsilateral supraclavicular fossa (SCF). The Axilla (Ax) and internal mammary nodal region (IMN) RT is uncommon due to the toxicities associated with them [6-10].

Radiation pneumonitis (RP) and lung fibrosis are two known toxicities that arise from incidental irradiation of adjacent ipsilateral lung in BC. Other toxicities include breast fibrosis, cardiac toxicity, skin toxicity and lymphoedema of the ipsilateral upper limb. The risk of cardiac toxicity in tangential radiotherapy treatment of left breast or chest wall is well studied in literature [6-8]. However most studies were pre-conformal CT-based planning and IMN irradiation was a common practice then.

RP and lung fibrosis is believed to represent the different ends in the clinical course of the disease rather than separate entities altogether. The natural history of the radiation lung injury can be divided into 5 phases: immediate phase (hours to days); latent phase; acute exudative/clinical RP phase (4–12 weeks post-RT); intermediate phase with resolution of exudate and deposition of fibroblast and the final phase when fibrosis is established (usually 6–12 months post-RT) [11-13]. Type II pneumocytes which produce surfactant are the cells associated with RP [13,14].

Literature on RP in breast irradiation is very heterogeneous: different simulation techniques (conventional fluoroscopy-based versus CT-based), different treatment planning systems [2-Dimensional (2-D) versus highly conformal CT-based], different sites treated (chest wall/breast +/− Ax/SCF/IMN) and also use of electrons to treat the chest wall +/− IMN after mastectomy.

McDonald *et al.*[11] reported in 1995, the incidence of radiological and clinical RP to be in the range of 27 – 40% and 0 − 10% respectively in patients with BC undergoing radiotherapy based on 2 studies in 1980. The details of the radiotherapy techniques and the grades of both radiological and clinical RP were not mentioned in that review. These figures are the most likely estimates of RP incidence in non-conformal planning days.

Currently there is a trend towards minimizing toxicity to organs at risks in the adjuvant treatment of breast cancer. The newer RT modalities or techniques used include Intensity Modulated Radiotherapy (IMRT), Tomotherapy, Accelerated Partial Breast Irradiation (APBI) and Intraoperative Radiotherapy (IORT). IMRT and Tomotherapy produce more conformal radiation delivery at the expense of increased integral dose. These newer modalities are more expensive and needs longer planning time. Smith BD *et al.*[15] has recently shown that the adoption of IMRT for BC in the United States have increased the cost of breast irradiation significantly.

In centers where there are limited resources or financial constraints, it is important to allocate the available resources accordingly without compromising the breast cancer patient’s treatment outcome. Hence the important questions are:

1. Which patient subgroups will benefit from these newer modalities of treatment in regards to early lung toxicity?

2. What are the dosimetric parameters, treatment factors and patient factors which predict for RP or early lung toxicity in adjuvant 3-Dimensional Conformal RT (3D-CRT) for BC?

## Methodology

Electronic databases were searched from 1995 till April 2011 (3D-CRT techniques would have been very unlikely before 1995) using the following inclusion and exclusion criteria for adjuvant RT studies in BC:

Inclusion Criteria:

The use of 3D-CRT.

Tangential photon fields or electrons for breast/chest wall treatment +/− LRRT.

Histology proven BC or Ductal/Lobular Carcinoma-in-situ (DCIS/LCIS).

Stage I-III BC.

Female.

Mastectomy or BCT.

Total dose of 50-60 Gy (or its equivalence in different dose fractionation).

Exclusion Criteria:

Male.

Non-3D-CRT techniques.

Stage IV BC.

Concurrent chemotherapy.

The use of Tomotherapy, APBI, interstitial implants, IORT or IMRT.

Inoperable BC.

Search was performed using PubMed search builder for Breast Radiotherapy AND Meta-analysis (or Systematic review); Breast Radiotherapy AND Toxicity; Breast AND Radiotherapy AND Pneumonitis. This produced 536 hits. Cochrane library, Google Scholar and reference sections of the major Radiation Oncology textbooks were searched to ensure no important studies were missed. Eight studies from these results were initially identified for the analysis and two further studies were identified by searching the reference sections of those 8 studies. Although four of the identified studies had overlapping data, different endpoints were used in the analysis [16-19]. Only one retrospective study was included [20].

Factors affecting the incidence and severity of RP were identified from the above studies and other thoracic irradiation studies (lung, oesophagus, lymphoma and others), which reported on RP. These were dosimetric parameters (V_dose_ and MLD), patient factors (smoking and age) and treatment factors (SCF and IMN RT; concomitant Tamoxifen and sequential chemotherapy).

Radiological RP grading criteria used include Arrigada, modified lung fibrosis CTC (common toxicity criteria) and Nishioka system [21]. The grouping of low grade radiological RP in this analysis are: Modified Arrigada grade 1 in Holli’s study [19] and Arrigada scores 1–3 in Goldman’s study [22]. Any grades or scores above these values are grouped as high grade radiological RP. The clinical RP is also divided into low and high grades with clinical RP grade 1 classified as low grade and clinical RP grade 2 or more grouped as high grade.

### Statistical methods

Data was analysed using Stata® software, version 11.0 (Stata Corp College Station, TX, USA), and level of significance set at 5%. To estimate the incidences of clinical RP and radiological RP, we pooled the values from each study, using the random effects meta-analysis model. As for the association between treatment-related factors and RP, we combined the individual effect sizes (Odds Ratios) using the random-effects model, using the method of DerSimonian and Laird, with the estimate of heterogeneity being taken from the inverse-variance fixed-effect model. Heterogeneity between studies was assessed by the chi-square test for heterogeneity as well as examining the i-squared statistic, which quantifies the level of heterogeneity. In the event of heterogeneity, we used the random effect model instead of the fixed-effect model to analyse the data. Publication bias for the primary endpoint (incidence of RP) was assessed via the Egger’s test.

## Results

### Incidence of RP

#### Radiological RP

**Figure 1:**
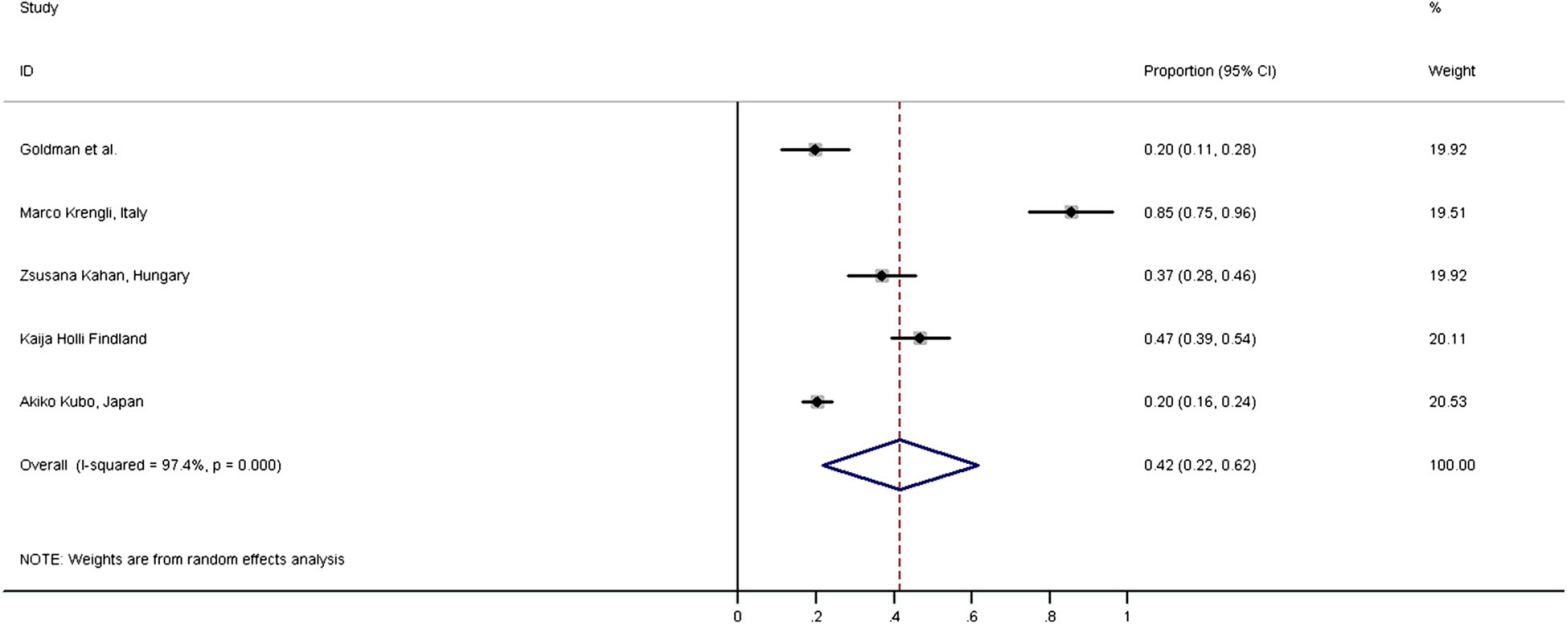
Meta-analysis: Incidence of radiological RP with 3D-CRT for BC in world literature.

The overall incidence of radiological RP in our meta-analysis [Figure 1] is 42% (95% CI = 22-62%) with large heterogeneity in the included studies (I-squared = 97.4%, p < 0.001) and Egger’s test did not show significant publication bias (p = 0.151). In the study by Marco Krengli *et al.*[23], the high detection rate of RP (85%) could be attributed to the use of high resolution CT images for post radiotherapy lung assessment. The incidence of RP was low, as reported by Akiko Kubo *et al.*[24] because only tangential RT fields were used and the RP assessment was mainly done by Chest X-ray (CXR).

The overall incidence of low grade and high grade radiological RP are 22% (95% CI = 17-27%) and 12% (95% CI = 9-33%) respectively. The I-squared heterogeneity index for low grade radiological RP is 1.7% (p = 0.313) and 97.5% (p < 0.001) for high grade.

#### Clinical radiation pneumonitis

**Figure 2:**
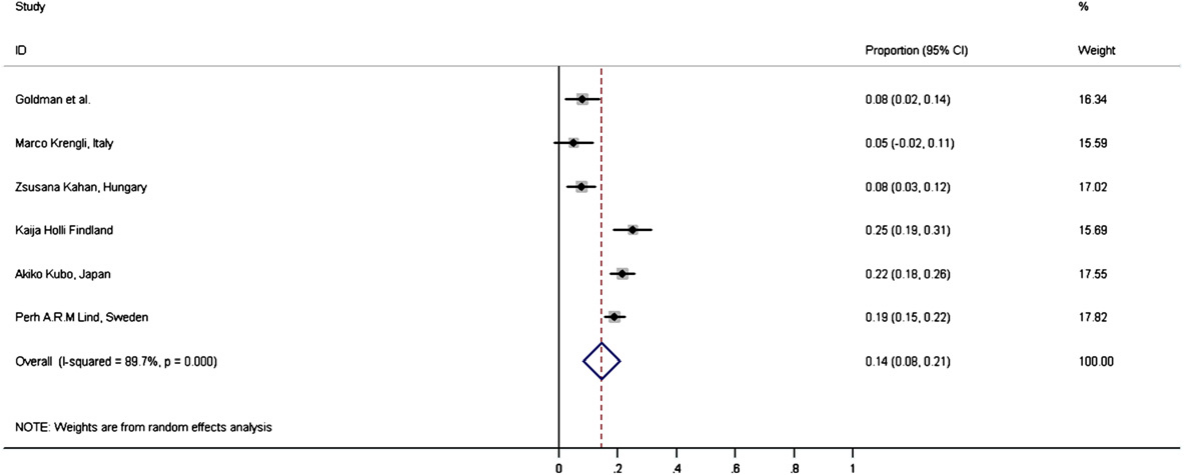
Meta-analysis: Incidence of clinical RP with 3D-CRT for BC in world literature.

The overall incidence of clinical RP [Figure 2] is 14% (95% CI = 8-21%) with large heterogeneity in the included studies (I-squared = 89.7%, p < 0.001). The Egger’s test did not show significant publication bias (p = 0.376). Either CTC2.0 or CTC3.0 assessment was used in the studies. There are no reported cases of CTC grade 4 or 5 toxicity in any of the studies.

The overall incidence of low grade and high grade clinical RP is 14% (95% CI = 9-18%) and 4% (95% CI = 2-7%) respectively. There is also large heterogeneity in the studies with I-squared = 79.3% (p = 0.002) and I-squared = 82.4% (p = 0.001) for low and high grade clinical RP respectively. The Egger’s test for publication bias is not significant for either low or high grade clinical RP (p = 0.855 and 0.407).

### DVH-related parameters

#### Volume of lung receiving a specified dose [V_dose_ ]

A systematic review by George Rodrigues *et al.*[25] in 2004 analysed 5 trials and reported a strong correlation between V_dose_ and RP in lung tumour irradiations. In contast, V_dose_ of the lung in breast or chest wall irradiation is likely to be smaller in value and hence we limit our analysis to studies reporting on V_20Gy_ to V_25Gy_ [Table 1] though some studies do report on other V_dose_ values.

**Table 1:** Relationship between dose volume parameters and early lung toxicity in 3D-CRT for BC in world literature

**Author, Year of data collection**	**Country**	**n**	**RP endpoint**	**Prognostic groups**	**Test type**	**p value**	**Comments**
Perh Lind *et al.* 1994*–*1998 [17]	Sweden	475	Clinical RP CTC-NCIC	Ipsilateral V_20Gy:_ ≈7%,≈20%, ≈30%,≈35%	2 sided Gamma statistics, G = 0.638	<0.001	Correlation between increasing V_20Gy_ to grade of lung toxicity. Assessed at 1,4 and 7 months.
Zsusana Kahan *et al.* 2001*–*2004 [31]	Hungary	119	Radiological RP – CTC 2.0	Mean Ipsilateral V_20Gy_ (%) (RP- vs RP+)	Student’s t-test (24.8% vs 31.1%)	0.005	3 and 12 months post RT CT-Thorax.
Javier Jaen *et al.* 2002 [44]	Spain	39	Change in perfused volume(%)	Bilateral V_20Gy_	Corr. Coefficient r = −0.414	0.026	6,12 and 36 months lung perfusion test.
Marco Krengli *et al.* 2002 – 2003 [23]	Italy	41	Radiological RP (G0-3)	Ipsi V_25Gy_	ANOVA	0.0007(G2 vs G0)	3 and 9 months post RT CT-Thorax.
						0.02 (G3 vs G0)	
Ulla Goldman *et al.* 2003 – 2005 [22]	Sweden	40	Radiological RP – Arigada score	Mean Ipsilateral V_20Gy_ (%) (RP- vs RP+)	24% vs 30%	-	4 months (CT-Thorax) and 5 months (CXR).
Akiko Kubo *et al.* 2005 – 2007 [24]	Japan	413	Radiological RP – CTC/AE 3.0	Ipsi V_20Gy_ ≤ 9.7%, Ipsi V_20Gy_ ≥ 9.8%	Cox regression RR = 0.67	0.13 (NS)	3 monthly CXR for 1 year. Only tangential whole breast irradiation.

*RP* radiation pneumonitis, *n* number of patients, V_dose_, volume of lung (%) receiving a certain dose (Gray), *CI* confidence interval, *CTC / CTC-NCIC* common toxicity criteria, *RT* radiotherapy.

Berit Wennberg *et al.*[16] analyzed 121 patients who had node-positive stage II BC, which was a subgroup from the main study by Perh Lind *et al.*[17]. He presented the data in the form of mean cumulative ipsilateral lung DVHs for four different treatment techniques. Data extracted from this paper showed that treatments techniques with V_20Gy_ ≤ 20% had a lower incidence of RP compared to V_20Gy_ > 20% (12.5% vs 28.4% respectively). Perh Lind *et al.*[26] also looked at a subgroup of 128 patients from his earlier paper [17]. He analyzed the data using the ROC (receiver operating characteristics curves), which showed the significance of ipsilateral V_20Gy_ in clinical RP (p = 0.008) and radiological RP (p = 0.009).

#### Mean lung dose (MLD)

MLD is also significantly correlated with the incidence and grade of RP, mostly in studies involving lung cancer or other thoracic irradiations [27-30]. However the data on MLD in BC is very limited [Table 2].

**Table 2:** Relationship between MLD and early lung toxicity in 3D-CRT for BC in world literature

**Author, Year of data collection**	**Country**	**n**	**RP endpoint**	**Test type,**	**p value**
Perh Lind *et al.* 1994*–*1998 [17]	Sweden	475	Clinical RP CTC-NCIC	Gamma statistics G = 0.669	<0.001
Zsusana Kahan *et al.* 2001–2004 [31]	Hungary	119	Radiological RP – CTC 2.0	Student’s t-test (RP + vs RP-)	0.003
Javier Jaen *et al.* 2002 [44]	Spain	39	Change in perfused volume (%)	Correlation coefficient, r = −0.447	0.013

*RP* radiation pneumonitis, *n* number of patients, *CTC/CTC-NCIC* common toxicity criteria, *RT* radiotherapy, *MLD* mean lung dose.

Though these studies used different end points, all of them showed significant impact of MLD on lung toxicity. As with the V_dose_, the study by Perh Lind *et al.* showed significant correlation between increasing MLD and the grade of lung toxicity using gamma statistics (p < 0.001) [17]. The MLD was 7.5 Gy, 13.5 Gy and 16.0-16.6 Gy for no RP, mild RP and moderate RP respectively.

In Zsusana Kahan’s study 20.5% of patients with radiological RP developed clinical symptoms. The MLD of patients with no RP versus RP in this study was 12.2 Gy vs 15.0 Gy respectively [31].

### Treatment factors

#### SCF irradiation

**Figure 3:**
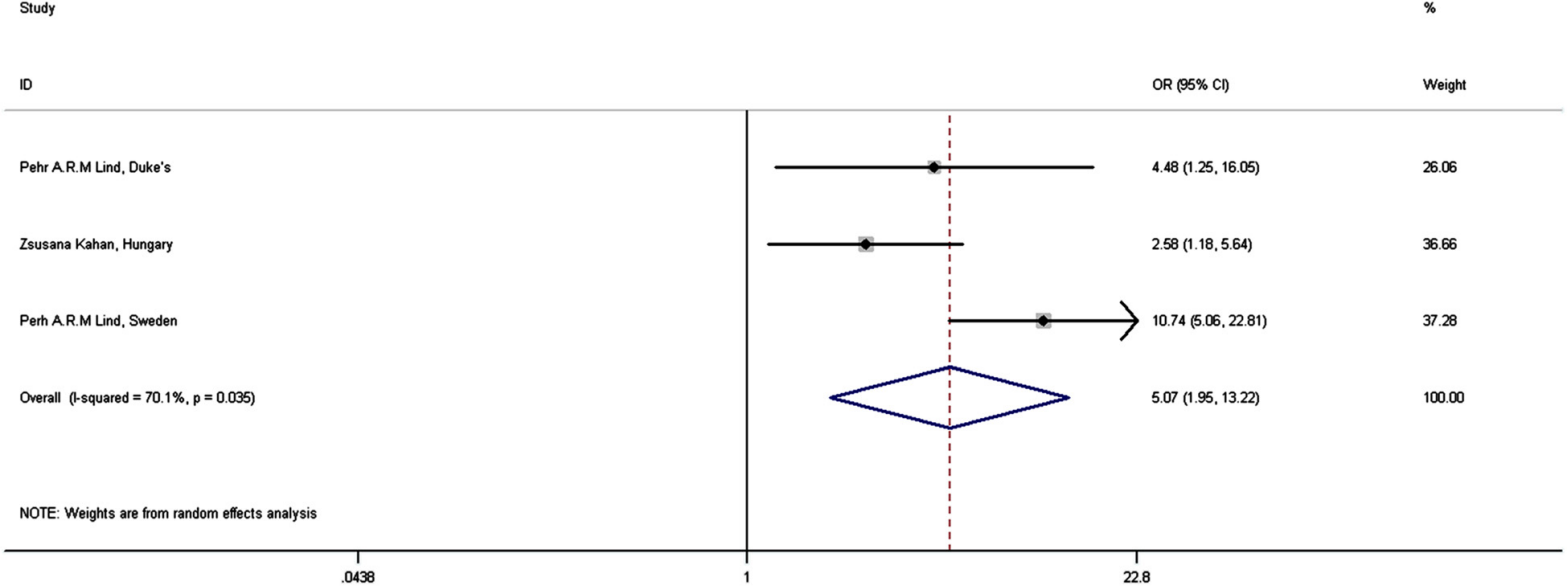
Meta-analysis: Effect of SCF irradiation on RP in 3D-CRT for BC.

Current meta-analysis shows a significant effect of SCF irradiation on the incidence of RP [Figure 3]. The odds ratio (OR) of having RP in this group is 5.07 (95% CI = 1.95-13.22). The heterogeneity between the studies (I-squared) is 70.1% (p = 0.035).

The subgroup analysis of 121 patients with node positive stage II BC by Berit Wennberg *et al.* also showed significant effect of SCF irradiation on RP [16].

#### IMN chain irradiation

**Figure 4:**
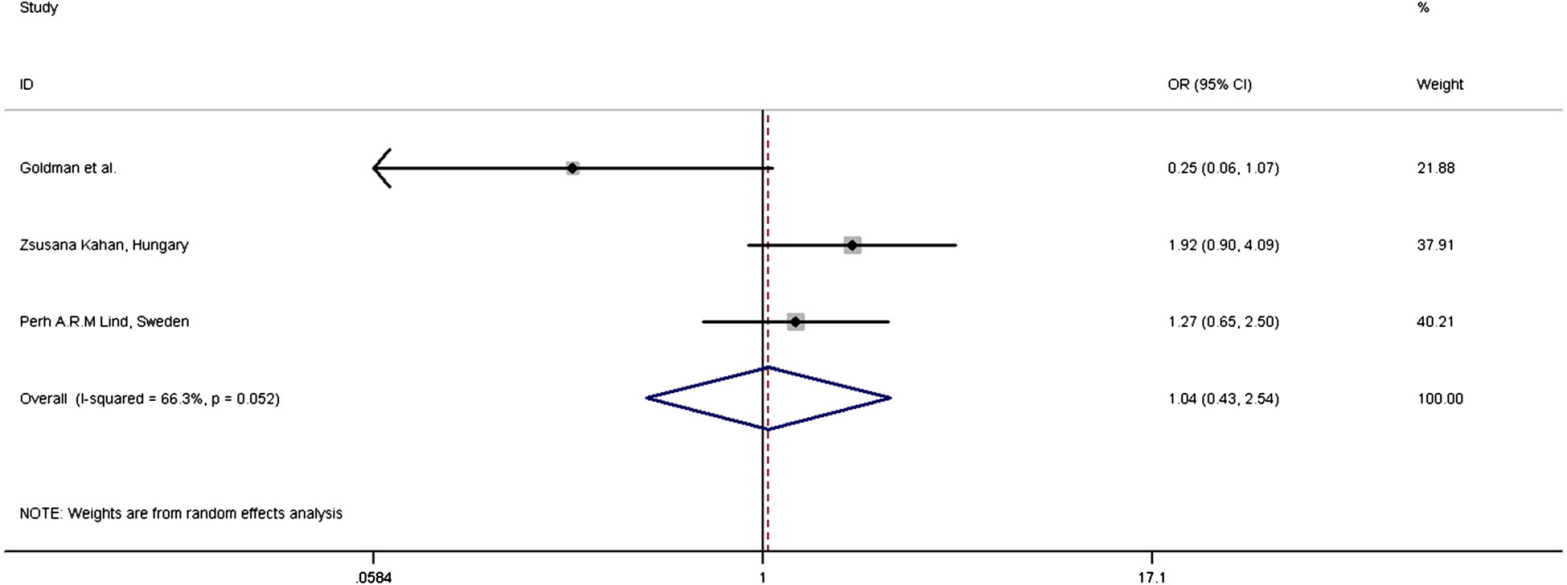
Meta-analysis: Effect of IMN irradiation on RP in 3D-CRT for BC.

Figure 4 shows that OR of having RP with IMN irradiation is 1.04 (95% CI = 0.43-2.54) and there is no statistically significant heterogeneity between the studies (I-squared = 66.3%, p = 0.052). The study by Goldman *et al.* had only 9 patients in the no IMN RT group [22]. The fields used in the IMN irradiation in the studies above vary significantly and they included oblique electron beam, anterior photon beam or deep oblique field.

#### Concomitant tamoxifen

The evidence of Tamoxifen causing lung fibrosis is so far unclear [32,33]. The OR of having RP with concomitant Tamoxifen use is 1.20 (95% CI 0.57 – 2.51) and heterogeneity index (I-squared) is 69.1% (p = 0.039).

#### Sequential chemotherapy-RT

Data on sequential chemotherapy and RP in breast irradiation is very scarce in the literature. Most studies looked at individual chemotherapeutic agents especially the taxane group [34,35]. The analysed studies are heterogeneous with multiple agents used, different doses and intensity of the chemotherapy. For example, Lind *et al.* (Sweden) [17] reported the use of CMF, FEC, dose intensified FEC and high dose chemotherapy with stem cell rescue. Kubo *et al.*[24] and Lind *et al.* (Duke’s) [20] did not mention the type of chemotherapy in their studies.

The OR of having RP in this group is 1.40 (95% CI = 0.44–4.50) with I-squared = 88.5% (p < 0.001) for heterogeneity of the included studies.

### Patient factors

#### Smoking

The effect of smoking on RP has been studied in a few trials involving thoracic irradiation. In two lung cancer studies [36,37], smoking has been associated with lower incidence of lung toxicity.

This positive effect of smoking is also seen in a trial by Leif Bjermer *et al.*[38] which looked at inflammatory response in breast cancer patients by bronchoalveolar lavage. Another large retrospective review by Silvia Johansson *et al.*[39] looked at 405 women who underwent radiotherapy for breast or oesophageal cancers also showed similar findings.

**Figure 5:**
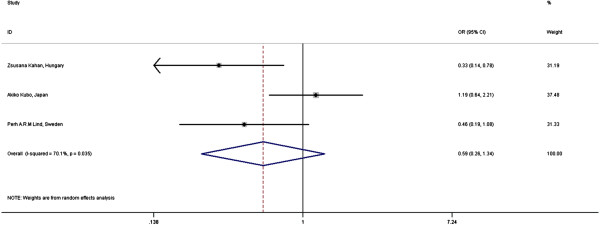
Meta-analysis: Effect of smoking on RP in 3D-CRT for BC.

Our analysis [see Figure 5] shows the OR of having RP in this smoking-group is 0.59 (95% CI = 0.26 – 1.34) with heterogeneity index (I-squared) of 70.1% (p = 0.035).

#### Age

Lung irradiation studies showed conflicting results of age in predicting for RP [28,36,40-42]. See BC studies in Table 3.

**Table 3:** Relationship between age and radiation pneumonitis in 3D-CRT for BC in world literature

Author, Year^*^	**Country**	**n**	**RP endpoint**	**Prognostic groups**	**Test type (results)**	**p value**	**Comments**
Lind *et al.* 1994*–*1998 [20]	Duke’s	613	Clinical RP, Grade > 2	Median age	Mann–Whitney test	0.64	RP > Grade 2 only (retrospective)
				(RP- vs RP+)	(54.9 yrs vs 55.2 yrs)		
Lind *et al.* 1994*–*1998 [17]	Sweden	180	Clinical RP CTC-NCIC	Mean age	Students t-test	<0.001	Loco-regional RT after mastectomy
				(RP- vs RP+)	(55.4 yrs vs 62.6 yrs)		
Kahan *et al.* 2001 – 2004 [31]	Hungary	119	Radiological RP – CTC 2.0	Mean age	Student’s t-test	0.012	-
				(RP- vs RP+)	(56.2 yrs vs 61.5 yrs)		
Jaen *et al.* 2002 [44]	Spain	39	Perfused lung volume (%)	≤55 yrs, >55 yrs	Box plot: ≤55 yrs vs >55 yrs	NS	Reduced perfusion in older age group
Kubo *et al.* 2005 – 2007 [24]	Japan	413	Radiological RP – CTC/AE 3.0	≤50 yrs,>50 yrs	Cox regression model	0.20	Only tangential whole breast irradiation
					(RR = 0.76, 95% CI 0.49 – 1.2)		

*RP* radiation pneumonitis, *n* number of patients, *CI* confidence interval, *CTC/CTC-NCIC* common toxicity criteria, *RT* radiotherapy, ^*^year of data collection, *NS* statistically not significant.

## Discussion

The early radiation induced pulmonary toxicity has been well studied in other thoracic irradiation, mostly involving lung cancers. Caution is needed in extrapolating the data in lung tumors to breast cancer patients. The demographics of the patients may differ significantly as can be seen in the proportion of smokers, age group, effect of the tumor itself on the lung function and also the location of the lung that is irradiated. Furthermore, the lung in BC patients is a healthy organ in contrast to lung cancer patients.

The major limitation that the authors faced in completing current analysis is the heterogeneity of the data in world literature, such as the clinical endpoints measurement, radiological and clinical RP grading system, different sensitivity in the detection tests of the radiological RP and the poor reporting of confounding factors in some studies.

Besides the dosimetric, treatment and patient factors mentioned earlier, other reported factors are pre-irradiation lung function and performance status, pre-existing respiratory diseases (chronic obstructive airway disease, interstitial pneumonitis, etc) and genetic predisposition. There is very little or no data on contribution of this factors in 3D-CRT for BC towards early lung toxicity and hence no analysis can be done.

### Radiological and clinical RP

The previous review by Mc Donald *et al.*[11] in 1995 have reported incidence of radiological RP in range of 27 – 40% and clinical RP in to be in the range of 0-10%. The overall incidence of radiological RP in current meta-analysis is 42% (95% CI = 22-62%) which is marginally higher compared to the previous review by Mc Donald *et al*. This may be due to the use of more CT scans in current studies.

Clinical relevance low grade or high grade radiological RP leading to late lung toxicity is not well known currently and is an area for further research not only in breast cancer patients but also other thoracic irradiations. Five studies in current analysis have shown poor correlation between radiological RP and clinical RP [18,19,22,31,43].

Fourteen per cent incidence of clinical RP in current analysis (95% CI = 8-21%), is also higher than the previous estimate by McDonald*.*. This can be due to more vigilant assessment by the physicians in current clinical trial settings. Furthermore, McDonald and colleagues only reported approximate values and the precise information was not always reported in the quoted studies. The late sequelae of low or high grade clinical RP is also not well known currently, as with radiological RP.

A subgroup analysis of 121 patients with stage II, node positive breast cancers by Berit Wennberg *et al.*[16] (from the large group of 475 patients in the study by Perh Lind *et al.*[17]) showed a slightly higher incidence of 23.1% clinical RP compared to 18.9% in the main study. This is probably due to the fact that the 475 patients recruited into the main study may include the mixture of very early stage disease who may not have had regional RT.

The reported incidences of clinical and radiological RP vary significantly between studies as seen in the test for heterogeneity (I-squared). This can be attributed to the different techniques, fields used and measurement variations of RP. Though the assessment of clinical RP is quite standardized using either CTC2.0 or CTC3.0 toxicity criteria, the threshold to diagnose clinical RP may vary significantly between physicians and between centers.

### DVH related dosimetric factors

The DVH related parameters (V_dose_ and MLD) are strongly associated with the clinical RP, radiological RP and change in physiologic lung functioning in the current analysis. The study by Akiko Kubo *et al.*[24] was insignificant most likely because of low ipsilateral V_20Gy_ of 9.6% (mean) and 18.8% (maximum).

In another study, Javier Jaen *et al.*[44] had shown that bilateral V_20_ was closely correlated to the change in perfused volume. However it is beyond the scope of this paper to discuss the merits of physiological or functional lung damage assessment.

It is difficult to deduce the exact threshold value of V_20Gy_ for clinical practice from this meta-analysis. However, other RT techniques or modalities can be considered, when ipsilateral V_20Gy_ > 30% [17,22,31]. Some clinical scenarios with high V_20Gy_ are very curved chest wall, bilateral breast radiotherapy and locally advanced BC. The ipsilateral V_20Gy_ should be kept below 24% if possible without compromising the required RT field coverage. The study by Akiko Kubo *et al.*[24] implies that further attempts at reducing ipsilateral V_20Gy_ below 20% may not be of any benefit.

There are other models in radiation induced lung injury that are not analyzed in this paper such as the normal tissue complication probability (NTCP) model and more advanced biologically based models [25,45,46]. There is very limited data on the use of these models in BC.

The large study of lung cancer patients by Kwa *et al.*[27] addressing RP have recommended the use of MLD or NTCP to predict the risk of RP. MLD is also found to be highly correlated to V_20Gy_ in lung irradiation studies [27,29]. The three studies quoted in this current analysis do show a strong relationship between MLD and early lung toxicity. Based on the studies by Perh Lind *et al.*[17] and Zsusana Kahan *et al.*[31], it should be safe if the MLD is limited below the range of 12-15 Gy to avoid serious lung toxicity in 3D-CRT irradiation for BC. Other techniques or modalities of radiotherapy may be considered if MLD exceeds 15 Gy.

### Treatment factors

SCF irradiation shows a strong association with RP incidence (OR = 5.07). The addition of SCF irradiation increases the amount of lung tissue irradiated and hence MLD and V_dose_[47,48].

The reported incidence of IMN and medial SCF LN metastasis is between 4-9% in axillary node negative patients and 16-52% in axillary node positive patients [49-52]. Despite the reported toxicities, positive outcomes from 2 studies by Overgaard M *et al.*[2,3] have renewed the interest in IMN irradiation as part of LRRT. Furthermore, modern conformal techniques are likely to reduce the heart dose. The current view of IMN irradiation may change depending on the outcome of EORTC trial 22922/10925 which completed accrual in 2004 and results are expected in 2012 [49]. The three years toxicity profile update of 4004 patients with IMN-medial SCF field irradiation reported a 4.3% lung toxicity rate in the IMN arm versus 1.3% in no IMN arm (p < 0.0001). There were no statistically significant difference between the two groups with regards to cardiac toxicity. Poorly powered study by Goldman *et al.* (9 patients in no-IMN RT group) [22] caused the non-significant findings in current meta-analysis.

Concomitant use of Tamoxifen has no statistically significant effect on RP in this meta-analysis, as in earlier studies [32,33]. Three large retrospective studies [53-55] examined the concomitant and sequential Tamoxifen in BC patients, and concluded that the use of both concomitant and sequential Tamoxifen was acceptable in terms of local relapse, distant metastasis and secondary malignancies. There was no statistically significant difference in lung toxicity between the groups.

The influence of concurrent chemotherapy-RT on RP incidence has been well studied in thoracic irradiation [11,28,56] and also two BC studies [11,57]. Concurrent chemotherapy-RT is not analyzed in current paper as it is seldom practised nowadays.

Data on sequential chemotherapy and RP in breast irradiation is very scarce in the literature [34,35]. Our result is not significant probably because of heterogeneous studies. Adjuvant Taxanes and Cyclophosphamide or biological agents (Bevacizumab) [58] have been reported to increase the risk of RP. Anthracyclines and Taxanes [59] are potent radiosensitizers. Cyclophosphamide have been reported to cause lung injury with or without the addition of RT in some case series [60]. Radiation recall can occur if Paclitaxel is used after RT and can cause pathological changes in the lungs at the previous RT field. Hence readers are encouraged to refer to data on individual drugs in the literature for clinical practice.

### Patient factors

There is a trend towards protective effect for smokers in breast irradiation in our meta-analysis. This effect was also reported in two other studies of thoracic irradiation [36,37]. This finding is quite puzzling because we expect that smokers with already damaged lung would be more susceptible to radiation induced lung injury. Some possible explanations are: 1) smokers may have higher threshold to develop clinical symptoms due to their already damaged epithelium; 2) the local immune reaction may not be as strong as non-smokers due to the damage to immune cells such as tissue macrophages in the lung epithelium causing reduced antibody secretion and exudates; 3) tissue hypoxia in smokers may have radio protective effect; 4) lung scarring in smokers may mask the appearance of RP on CT scans.

In current analysis, there is a conflicting effect of age on both radiological and clinical RP in 3D-CRT for BC. The studies by Akiko Kubo *et al.*[24] and Perh Lind *et al.* (Duke’s) [20] did not show statistically significant effect of age. In the former study, only tangential whole breast irradiation was used and the volume of irradiated lung was very small. There could have been significant bias in retrospective data in the latter study.

Based on the other three studies, age > 55 yrs is a risk factor for RP in 3D-CRT for BC. However, the long term effect of early lung toxicity is not known and may take many years before it becomes clinically significant, especially in the younger age group.

Another type of early lung toxicity reported in the literature but not discussed here is the radiation-induced bronchiolitis obliterans organizing pneumonia (BOOP) syndrome in which the lung injury occurs outside the radiation field or even in the contra-lateral lung. Kubo *et al.* have reported 2.9% incidence of BOOP syndrome with all cases of radiological RP ≥ grade 2 developing BOOP syndrome. A survey in major hospitals in Japan from August 1999 to March 2000 showed an incidence of 1.8-2.19% in patients radiated after BCT [24,61].

## Conclusion

The DVH-related parameters (V_dose_ and MLD) and SCF irradiation are the strongest parameters associated with RP. Attempt should be made where possible to keep the ipsilateral lung V_20Gy_ < 24% and MLD < 15 Gy without compromising the required radiotherapy coverage. Other RT techniques can be considered when ipsilateral lung V_20Gy_ >30 Gy or MLD > 15 Gy. Other factors that increase the risk of RP are age >55 yrs and probably IMN irradiation. However, caution is needed in treating younger age group due to the possibilities of late sequealae.

Concomitant Tamoxifen do not increase the risk of developing RP. Readers are encouraged to search the data on individual chemotherapeutic agents for clinical practice. Though smoking is noted to have protective effect, this can just be a masking effect rather than physiological protection against radiation induced damage.

Biochemical markers can be another factor to predict for early lung toxicity in 3D-CRT for BC. The combination of elevated transforming growth factor (TGF)-beta1 levels during RT and MLD has been reported as having predictive value in non-small cell lung cancers [62]. This together with use of radioprotectors such as Pentoxifylline can be an area for further research in RP [63].

## Consent

No individual patient consent was required for the publication of this manuscript as it only involves analysis of published trials.

## Competing interests

The authors declare no conflict of interest. The authors alone are responsible for the content and writing of the paper.

## Authors’ contributions

All authors’ contributed equally for the work in this manuscript. GK and WLC was involved in the initial proposal writing and was later joined by EA in the database search process. Thereafter, extracting the raw data from the publications were done by GK and WLC. EA involved mostly in the statistical analysis for the manuscript. All three authors actively participated in the final write-up and also iterative correction of this manuscript. All authors read and approved the final manuscript.
